# Draft Genome Sequence of the Naphthalene Degrader *Herbaspirillum* sp. Strain RV1423

**DOI:** 10.1128/genomeA.00188-14

**Published:** 2014-03-20

**Authors:** Ruy Jauregui, Belén Rodelas, Robert Geffers, Nico Boon, Dietmar H. Pieper, Ramiro Vilchez-Vargas

**Affiliations:** a Microbial Interactions and Processes Research Group, Helmholtz Centre for Infection Research, Braunschweig, Germany; b Departamento de Microbiología, Facultad de Farmacia, Universidad de Granada, Granada, Spain; c Genome Analytics, Helmholtz Centre for Infection Research, Braunschweig, Germany; d Laboratory of Microbial Ecology and Technology (LabMET), University of Ghent, Ghent, Belgium

## Abstract

*Herbaspirillum* sp. strain RV1423 was isolated from a site contaminated with alkanes and aromatic compounds and harbors the complete pathway for naphthalene degradation. The new features found in RV1423 increase considerably the versatility and the catabolic potential of a genus of bacteria previously considered mainly to be diazotrophic endophytes to plants.

## GENOME ANNOUNCEMENT

Bacteria of the *Herbaspirillum* genus are commonly described as diazotrophic plant endophytes (1, 2), although recent studies show their greater versatility. *Herbaspirillum* spp. have been found in coastal hydrothermal fields (3), ground water (4), drinking water distribution systems (5), and human sources, including wounds, respiratory tract, gastric juice, feces, urine, and samples from otitis, bacteremia, and cystic fibrosis patients (6–8). Moreover, several studies provided insights into the potential role of *Herbaspirillum* spp. in the biodegradation of fluoranthene (9) and 4-chlorophenol (10). Even though the versatility of *Herbaspirillum* spp. becomes more and more evident, to date, all but one genome project (7) have been focused on root-associated endophytic bacteria, while the genomes of *Herbaspirillum* spp. with catabolic potential remain unexplored.

Here, we present the draft genome sequence of *Herbaspirillum* sp. strain RV1423, isolated from the underground water of a hydrocarbon-contaminated environment (11, 12).

The genome was sequenced using the Illumina MiSeq platform, which generated 8,023,213 paired-end reads of 250 bp assembled using Edena (13, 14), producing 131 contigs with a total genome size of 6.21 Mb (59% G+C content; *N*_50_, 129 kbp; mean, 47.41 kb) and an average 300-fold coverage. Automatic annotation was performed using the RAST server version 4.0 (15), generating 5,732 features potentially assigned to protein-coding genes (open reading frames [ORFs]).

Although the 16S rRNA gene sequence shows 99% similarity with that of *Herbaspirillum lusitanum* P6-12, they share only 2,760 ORFs with >80% similarity (amino acid sequences), and 1,472 ORFs observed in the genome of strain RV1423 are absent from the genome of strain P6-12.

Strain RV1432 harbors genes encoding all the enzymes necessary for the mineralization of naphthalene via the gentisate pathway. Only four *Herbaspirillum* genomes (those of *H. lusitanum* P6-12 [16], *H. seropedicae* Os34 [17] and Os45 [18], and *H. huttiense* subsp. *putei* IAM 15032 [19]) harbor two gentisate dioxygenase genes, and RV1432 harbors four gentisate dioxygenases.

Comparing the draft genome of strain RV1423 and those of *Polaromonas naphthalenivorans* CJ2 (20) and *Ralstonia* sp. strain U2 (21), we found high similarities in the genomic regions involved in naphthalene degradation, suggesting that strain RV1423 uses the *nag* pathway (20).

Strain RV1432 harbors two independent *nag* operons, one displaying identical genomic organization and similarity to that previously described in strain CJ2. The second *nag* operon is a fusion between the *nag* operons of strains CJ2 and U2. The fusion occurs in the gene encoding a 2-hydroxychromene carboxylate isomerase. The first 12 genes of the operon show >99% similarity to those of strain CJ2, while the 5 distal genes show >99% similarity to those of strain U2.

A comparison between the draft genome of RV1423 and the 10 genomes/draft genomes of the strains *H. seropedicae* SmR1 (22), P6-12 (16), *Herbaspirillum frisingense* GSF30^T^ (2), *Herbaspirillum* sp. GW103 (23), Os34 (17), Os45 (18), IAM 15032 (19), CF444, YR522 (3), and JC206 (7) showed that all strains harbor genes for ammonium assimilation and cobalt-zinc-cadmium resistance. The genes for denitrification were found only in strains RV1423, GSF30^T^, Os34, Os45, and SmR1. Genes for nitrate and nitrite ammonification were found in all strains but JC206. The genomes of strains RV1423, P6-12, and GSF30^T^ encode carbon monoxide oxidases, and mercury reductases were found only in strains RV1432 and GW103. Strain RV1423 harbors an alkylmercury lyase that is 94% similar to YP001102036 of *Salmonella enterica* subsp. *enterica* serovar Newport SL254 (24).

### Nucleotide sequence accession numbers.

This whole-genome shotgun project has been deposited in the EMBL-EBI European Nucleotide Archive (ENA) under accession no. CBXX010000001 to CBXX010000131.
